# Navigating Knowledge: Effects of State Curiosity on Children's Word Learning and Information Seeking

**DOI:** 10.1111/desc.70226

**Published:** 2026-05-19

**Authors:** Anna Caunt, Shaun Dordoy, Rana Abu‐Zhaya, Alastair Smith

**Affiliations:** ^1^ School of Psychology University of Plymouth Plymouth UK; ^2^ Brain Research & Imaging Centre University of Plymouth Plymouth UK; ^3^ Division of Psychology and Language Sciences University College London London UK

**Keywords:** curiosity, exploration, naturalistic learning, real‐world search, state curiosity, word‐learning

## Abstract

Early learning takes place within complex, multimodal environments where curiosity plays a critical role in guiding exploratory behaviors and supporting cognitive development. While trait curiosity shapes children's early learning experiences, state curiosity is context‐dependent fostering early learning, in‐part, through information seeking behaviors. Here, we explore whether state‐induced curiosity can influence exploratory behaviors and facilitate word‐learning within a multimodal search task that mirrors real‐world learning environments. Children (2.5‐ to 4‐year‐olds) will be introduced to novel objects and labels via a storybook. After exposure to each novel object, participants will locate the target object (i.e., a sticker depicting the novel object), hidden within one of ten boxes uniformly distributed across a real‐world space. Unbeknownst to participants, the target will only be hidden within a single hemispace. In the “High‐Curiosity” condition, the storybook should elicit greater curiosity, whereas the “Low‐Curiosity” condition will present novel words in a context that should invoke lower levels of curiosity. Following the search task, participants will complete a forced‐choice word learning task. Findings will provide insights into whether and how curiosity facilitates word learning in rich, multimodal contexts and how this relationship can be impacted by children's explorative strategies, including their sensitivity to environmental statistics.

**Summary:**

- Curiosity drives early development, but the extent to which it facilitates learning within a multimodal environment remains underexplored.
- The study examines how high versus low induced curiosity states influence toddlers' exploratory behavior, including search efficiency and adaptation to spatial probability cues.
- The study assesses whether higher state‐induced curiosity leads to stronger retention and recognition of novel word‐object mappings compared to the lower curiosity condition.
- The study examines how curiosity‐driven search strategies relate to novel word learning and whether learning is moderated by how children choose to search for information.

## 1 Introduction

Curiosity is a cornerstone of cognitive development, driving children's exploration and learning throughout development (Chouinard et al. 2007; Shouse et al. 2007; Jirout et al. 2024; Jirout and Zimmerman 2015; Oudeyer et al. 2016). According to Loewenstein's (1994) information‐gap theory, curiosity arises from an intrinsic desire to seek out information and close knowledge gaps without the need for external reward; resolving uncertainty is inherently rewarding (Berlyne 1960, 1971), reinforcing further information‐seeking behaviors. For instance, by 16 months, infants use pointing gestures to obtain information on how novel objects function (Begus and Southgate 2012; Begus et al. 2014). This innate drive for exploration manifests in various ways, including asking information‐seeking questions (Ronfard et al. 2018) and engaging in exploratory play (von Hofsten 2018). During play, preschoolers often focus on toys with mechanisms they do not yet understand, befitting their natural curiosity about cause‐and‐effect relationships (Schulz and Bonawitz 2007). Further, infants as young as 11 months old preferentially explore information that leads to unexpected outcomes (Stahl and Feigenson 2015), suggesting that violations of expectations promote further exploration and learning. Such activities are guided by infants’ neophilic tendency to focus on novel and complex stimuli (Gopnik 2020). As infants physically explore their environment and interact with new objects, caregivers often respond with verbal input, such as labeling objects the infant is engaging with (Custode and Tamis‐LeMonda 2020). This multimodal aspect of early learning, where infants simultaneously engage in an action (e.g., shaking a rattle) while receiving language input (e.g., hearing the words “rattle” and “shake”), creates optimal conditions for word learning (Suarez‐Rivera et al. 2022).

Yet, despite extensive research on curiosity and active learning, evidence that curiosity‐driven exploration enhances word learning is mixed. Active selection does not reliably enhance word learning outcomes when infants must independently manage information selection in touchscreen tasks (Ackermann et al. 2020; Bothe et al. 2026), or in gaze‐contingent learning paradigms (Bazhydai et al. 2026). Similar null effects have been observed in children aged 3–8 years, where granting learners control over which objects to inspect, and for how long, did not improve word learning accuracy (Zettersten and Saffran 2021). Yet, other studies suggest that active engagement can scaffold word learning outcomes under more constrained or supportive conditions. These effects do not arise from active control per se, but from forms of self‐directed exploration in which the learner's actions are more tightly coupled to informative input or reduced decision demands. For example, self‐directed exploration improves recognition of newly learned word‐object mappings in preschoolers when choice spaces are structured or feedback is available (Partridge et al. 2015), and active control enhances toddlers’ recognition of labeled stimuli in paradigms that limit the need to independently identify informative exploration targets (Li et al. 2025).

The equivocality could be explained by the differences in task design. In many active‐learning paradigms, children must select what to explore and translate that exploration into explicit responses, which may increase cognitive demands (Ackermann et al. 2020). Consistent with this view, child preferences for active over passive learning appear to depend on how task demands are structured (Ackermann et al. 2024). Thus, null effects of active exploration may reflect situations in which the cognitive demands of information selection outweigh benefits, whereas positive effects may emerge when exploration is more tightly aligned with learning‐relevant input. Moreover, most existing work examines curiosity‐driven information selection in constrained contexts, leaving open the question of how curiosity operates during early word learning in dynamic environments that more closely resemble infants’ real world learning experiences. As a result, it remains unclear whether active control improves word learning outcomes, highlighting the importance of identifying the conditions under which curiosity‐driven exploration supports learning. Indeed, curiosity's impact on learning may be best captured in multimodal designs that integrate different types of information across various contexts (Bazhydai et al. 2026).

### 1.1 Trait and State Curiosity

Addressing how curiosity supports word learning in naturalistic contexts requires accounting for curiosity both as a momentary state, that is, *state curiosity*, which is elicited by the learning environment in specific situations, and as a stable personality characteristic, that is, *trait curiosity*. This distinction is particularly relevant when considering how uncertainty, ambiguity, and novelty may be experienced differently across developmental stages. Trait curiosity reflects a readiness to explore novelty and a tolerance of uncertainty (Grossnickle 2016; Loewenstein 1994), shaping what children find curiosity‐evoking. In contrast, state curiosity emerges in response to specific contexts and is often triggered by uncertainty. State and trait curiosity are interdependent and closely linked with information seeking behavior, such that children with higher trait curiosity may be more likely to experience elevated states of curiosity in response to learning opportunities (Jirout et al. 2024). In exploring the importance of curiosity for learning, Walin et al. (2016) asked 7‐year‐old children to rate their curiosity about 34 trivia questions. After a training phase and controlling for prior knowledge, children more accurately recalled answers to questions they rated higher on the curiosity scale. Similarly, in 2‐ to 4.5‐year‐old children, uncertainty appears to drive exploration: when children searched for a hidden animal on a tablet‐based experiment, they persisted longer when the target was unknown compared to when it was known (Ruggeri et al. 2023). Furthermore, knowledge gaps appear to shape exploration across development: 6‐ to 9‐year‐olds preferentially sampled objects whose label they reported not knowing, whereas 5‐year‐olds did not (de Eccher et al. 2024).

These findings suggest that knowledge gaps or uncertainty can motivate information‐seeking behavior in childhood (de Eccher et al. 2024; Ruggeri et al. 2023; Walin et al. 2016). However, the extent to which uncertainty guides children's exploration appears to vary with development. Recent work suggests that sensitivity to uncertainty may emerge at younger ages in tasks that place lower demands on memory and decision making, and only at older ages does it extend to tasks that require maintaining and acting on internally represented knowledge gaps (Coughlin et al. 2015; de Eccher et al. 2024; Hembacher et al. 2014). Thus, developmental differences in uncertainty‐driven information seeking may reflect variation in how effectively uncertainty elicits momentary states of curiosity. State curiosity motivates engagement and learning in response to situational cues (e.g., curiosity‐invoking questions), sparking further interest in a topic and improving learning outcomes (Ligneul et al. 2018; Marvin and Shohamy 2016). This is, however, also moderated by an individual's propensity to fill an information gap or relieve uncertainty, which may account for variation in the effectiveness of inducing state curiosity.

Berlyne's (1960, 1971) theoretical account proposes that curiosity is elicited through situational components designed to increase arousal and motivate information seeking behavior—for example, novelty, uncertainty, ambiguity, and violation of expectation. Empirical work has demonstrated that such conditions promote exploration, persistence, and learning in infancy, particularly when outcomes are unexpected or partially unknown (e.g., Kidd and Hayden 2015; Stahl and Feigenson 2015). From this perspective, state curiosity may be systematically manipulated by structuring learning tasks to preserve information gaps, introduce uncertainty or violate expectations. Trait curiosity, detectable in infancy (Altmann et al. 2025), can explain individual differences in the induction of state curiosity (Lee et al. 2023). Specifically, a caregiver questionnaire for children aged 2–5 years old assesses multiple facets of curiosity: novelty‐seeking, investigative behaviors, and information seeking behaviors (Altmann et al. 2024). Such measures enable assessment of stable individual differences in curiosity and allow researchers to examine whether such differences shape children's responsiveness to curiosity‐eliciting situations like situational knowledge gaps and uncertainty.

### 1.2 The Interrelation Between Learning and Information‐Seeking Behaviors

Early learning occurs within multimodal, multisensory social settings (Goddu and Gopnik 2024; Gopnik 2020). In infants’ successful navigation of these settings, early learning is revealed to be a complex non‐linear process wherein motor, cognitive, and language development interact dynamically. As infants gain dexterity and independence, new pathways for communication and skill acquisition unfold. Dynamic systems theory provides a framework for understanding this process (Smith and Thelen 2003). For example, when an infant reaches for an object, tactile and visual feedback support learning about its properties. Such feedback is key for developing perceptual‐motor skills, as the action of reaching is not only a physical skill but an opportunity to integrate sensory information to form a more complete understanding of the object (Smith and Thelen 2003). This is evident in object play, which supports object‐specific knowledge and language skills (Kidd et al. 2012; Schulz and Bonawitz 2007). For instance, as an infant reaches for a ball, they are not only learning about the ball's physical properties but also forming an association with the word “ball,” which is likely to be produced around them as they interact with the ball. These interactions are further shaped by state and trait curiosity, which motivate children to explore their surroundings, therefore creating opportunities for multisensory learning. By guiding attention toward novel or uncertain stimuli, curiosity supports infants in connecting sounds, objects, and actions, thereby facilitating both language and cognitive development (Twomey and Westermann 2018).

Since early language learning occurs in multimodal environments, it would be best studied in contexts where infants can engage multiple senses when exposed to novel items. Yet, most word learning studies employ experimental paradigms that differ substantially from how infants encounter novel object‐word associations in everyday life. In typical word‐learning tasks (e.g., Ellis et al. 2015), infants are usually familiarized with a novel object‐word pairing by seeing an image of a novel object and hearing its novel label, for example, “Wug.” Infants are then presented with two side‐by‐side images, one familiar and one unfamiliar, while hearing an experimenter ask, “Where is the Wug?” (Ellis et al. 2015). Infants’ success in learning the word is measured by how long they look at the correct object or how quickly they shift their gaze toward the correct object once they hear the object label. Other word learning studies are slightly more naturalistic (Piazza et al. 2021; Smith et al. 2002). In Smith et al. (2002), for instance, researchers used weekly play sessions to teach toddlers novel word‐object pairings and then tested toddlers’ abilities to generalize the newly learned names to new object exemplars. While such studies offer useful insight, they focus on how adults (or pre‐recorded voices) introduce novel words to infants and do not account for children's role in actively co‐constructing their own learning of novel words through self‐directed exploration. Thus, approaches emphasizing self‐driven, goal‐directed exploration could provide deeper insights into multimodal knowledge acquisition, particularly mapping novel objects to their referents.

### 1.3 The Role of Search and Exploration

Children's exploratory behaviors are guided by both curiosity‐driven information seeking (Gopnik 2020) and an emerging ability to detect multimodal probabilistic patterns in their environment (Clerkin et al. 2017; Clerkin and Smith 2022). As children interact with their surroundings, they integrate statistical regularities with prior knowledge to optimize learning and decision‐making (Denison and Xu 2014; Kushnir and Gopnik 2007). This capacity allows them to develop adaptive search strategies, such as in probability cueing, where learned probabilities direct attention and actions (Denison and Xu 2014; Gweon et al. 2010; Kushnir and Gopnik 2005; Ruggeri et al. 2019). For instance, in locating a hidden toy, children adjust their search behavior based on past experiences, demonstrating an implicit understanding of the statistical properties of their surroundings (Hartley et al. 2021). Environmental statistics guide predictions and information search (Denison and Xu 2014; Gweon et al. 2010; Kushnir and Gopnik 2005), and facilitate language acquisition (Griffiths et al. 2010; Saffran and Kirkham 2018; Smith et al. 2018). Detecting and adapting to these regularities is central to effective learning (Ruggeri 2022; Ruggeri and Lombrozo 2015; Ruggeri et al. 2017).

Crucially, curiosity‐driven exploration is mediated not only by novelty but also by expected information gain (Poli et al. 2024). Infants engage in distinct patterns of exploration when searching for information (Altmann, Bazhydai, Karadağ, et al. 2025; Altmann, Bazhydai and Westermann 2025), and by 24 months of age, they adapt their information search to task specific demands, demonstrating unique exploratory strategies (Poli et al. 2024; Vaisarova et al. 2024). More broadly, Hills et al. (2006) proposed that learning, memory, and attention, are guided by foraging‐like control mechanisms (Smith and De Lillo 2022; Talbot et al. 2022): language learning in particular entails actively sampling environmental information and forming associations based on probabilistic patterns—processes that mirror adaptive foraging strategies. Within multimodal social environments, children selectively explore objects and evaluate cues that maximize their information gain, reflecting efficient navigation of complex learning environments (Smith et al. 2018). We propose that these combined capacities facilitate word learning, and we advocate for designing tasks that maximize the relationship between learning and active information search.

### 1.4 The Current Study

We will explore how state curiosity influences children's novel word learning through active environmental search. Studies show that epistemic curiosity is evident early in development (Begus and Southgate 2012; Begus et al. 2014; Stahl and Feigenson 2015), and that uncertainty‐driven information seeking varies throughout development emerging later for some learning tasks (de Eccher et al. 2024; Ruggeri et al. 2023). In the current study, we will focus on children between 2.5 and 4 years old—a period characterized by rapid lexical growth (Clark 2019) during which word learning becomes more flexible: for example, while 2.5‐year‐olds show context dependent generalization, 3‐ and 4‐year‐olds are increasingly able to generalize words across contexts (Vlach and Sandhofer 2011). Thus, between 2.5 and 4 years, children's increasingly intentional and coordinated information‐seeking behaviors (Ronfard et al. 2018), may become better aligned with the demands of word learning.

Children will be exposed to four novel word‐object associations presented in a storybook. Object‐word pairings will be introduced sequentially, with each exposure followed by a search task where children must locate the target object hidden inside one of 10 boxes evenly distributed across two distinct hemispaces. Targets will appear only within a single hemispace, allowing us to assess whether children adapt to the statistical regularities of the search environment. To examine the effects of curiosity, participants will be divided into two groups. In the “High‐Curiosity” (HC) condition, children will experience a narrative designed to induce curiosity, reflecting Berlyne's (1960) collative properties. The narrative emphasizes uncertainty and ambiguity by presenting children with a task with an unknown goal, that is, finding parts to help an alien friend build an unknown object. Prompts are embedded throughout the narrative to highlight information gaps (e.g., “I wonder what this part is for”), and novelty is emphasized through repeated references to the unfamiliar appearance of the parts during exposure (e.g., “I don't think I've ever seen anything that looks like that before!”). In addition, an event violating participants’ expectations is incorporated into the narrative, while the search space itself will be structured with a moderate amount of complexity, including decorations and lighting reflecting the story. Conversely, the “Low‐Curiosity” (LC) group will encounter the four novel object‐word pairs without the accompanying narrative and will search within a featureless space. Following the search task, children will complete a forced‐choice word recognition task. By comparing learning outcomes across curiosity conditions, this exploratory study will investigate how curiosity‐driven search strategies enhance novel word learning. We hypothesize that: (1) higher state‐induced curiosity (compared to the LC condition) will improve novel word learning, with the relationship moderated by participant age and trait curiosity; (2) higher state‐induced curiosity will lead to more efficient search behavior, also moderated by participant age and trait curiosity; and (3) more efficient search strategies will be associated with better word‐learning, moderated by higher state‐induced curiosity and age.

## 2 Methods

All study materials can be found on the project OSF page, alongside a feasibility report: https://osf.io/5bmhk/overview?view_only=dc3d7975debb4c2bbfa6060d428f373a. We also include a document with further demographic details about the testing location.

### 2.1 Participants

To determine the required sample size, a power analysis was conducted on simulated data following recommendations set out by Kumle et al. (2021) using the simr package (Green et al. 2016). Power was estimated to detect a main effect of Condition (i.e., curiosity) on word learning accuracy (correct/incorrect) using a Binomial Generalized Linear Mixed Model (See Section 2.5). Accuracy in the LC condition was set to 30% and set to 40% for the HC condition. Participant variability was included as a random intercept with a moderate standard deviation (i.e., SD = 0.4). Age and trait curiosity were included as covariates in the model, although they were set to 0 to allow for a conservative estimation of power that did not assume any additional variance explained. An alpha level of 0.05 was selected using 1000 iterations. Results indicated that a sample of 90 participants would be sufficient to provide adequate power (i.e., mean power = 94.60%, 98% CI = [93.01, 95.02]), 45 per condition, equally divided across three age groups (2.5–3, 3–3.5, and 3.5–4 years‐old). Code used to replicate this power analysis is available on the OSF page.

Families will be recruited through the database of an established developmental research laboratory, located in a small seaside urban area in the United Kingdom (population: 277,695). Only 16.8% of the city's working‐age population falls into the highest social grade, compared to 23.5% nationally, while 48.6% are in lower socio‐economic grades, compared to 43.7% across England and Wales (ONS 2023). Moreover, the city is less diverse than other areas of the United Kingdom with the white population making up 95.9% of the city, compared to 81.7% in England and Wales (ONS 2023).

Using the standard lab questionnaire (available on the OSF), we will collect the following demographic information: the child's age and sex, parents’ nationality, place of birth, occupation, and highest educational qualification. Participants will receive a small gift in line with lab procedures. Ethical approval has been granted by the institution's Research Ethics Committee and written informed consent will be obtained from caregivers prior to participation.

### 2.2 Apparatus

**FIGURE 1:**
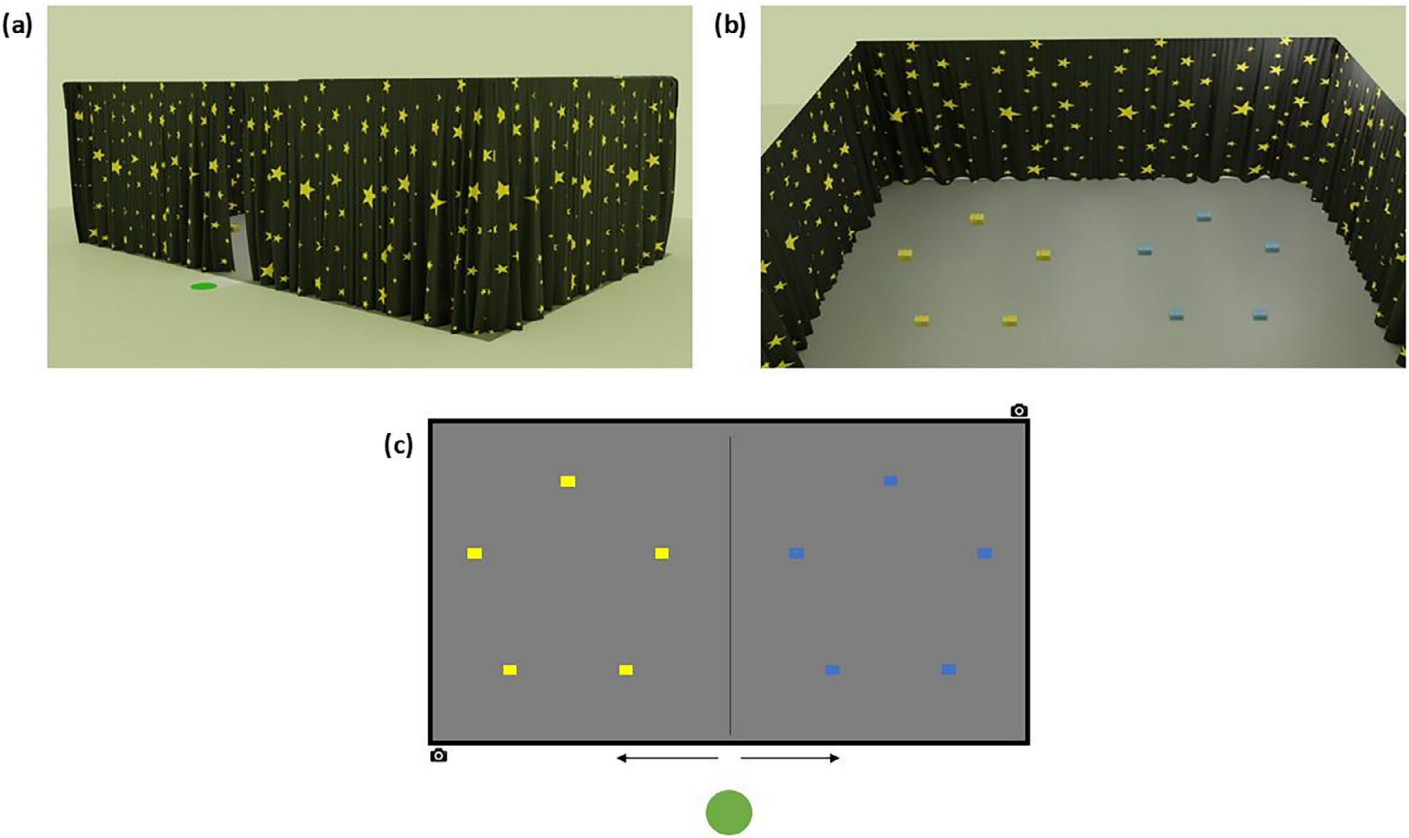
The experimental environment. Virtual render of the planned experimental set up. (a) A view from the outside of the experimental setup; (b) view of the experimental setup; and (c) a top down, to‐scale schematic of the search array. The green circle represents the location each participant will begin their search and the accompanying arrows indicate the location where encompassing curtains will be separated to allow access to the search array. Each blue/yellow rectangle represents a search location (i.e., box). Two camera symbols represent the approximate location of the cameras filming the experimental session.

**FIGURE 2:**
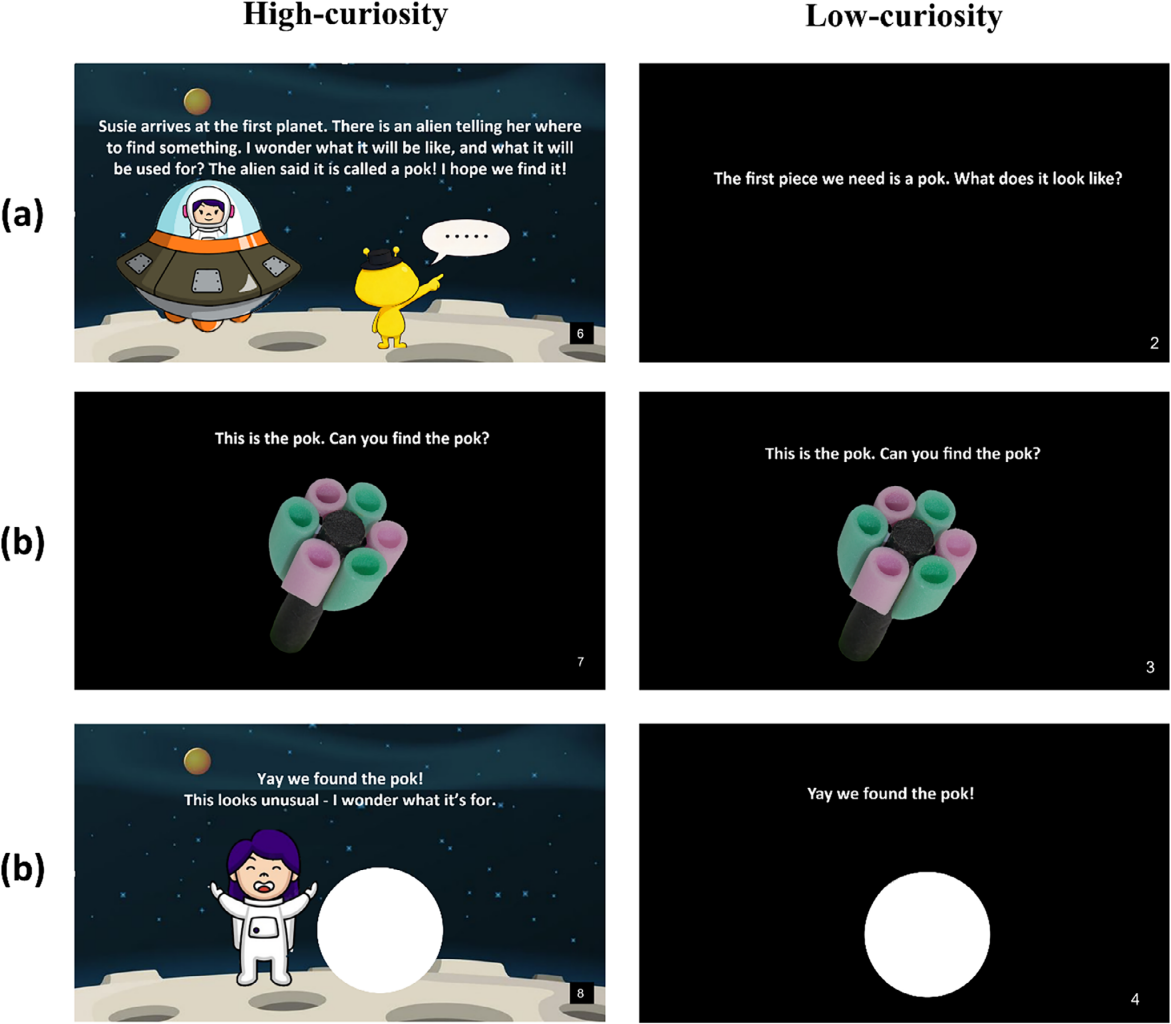
Novel object and word introduction in both the High‐curiosity and Low‐curiosity storybook. Example pages extracted from the storybooks used in the HC and LC conditions. (a) The point at which the child is first introduced to the novel word (e.g., pok) in the story; (b) the novel object and novel word are paired, and the child is prompted to begin the search task; and (c) the child returns to the story to fill in the sticker slot.

The study will be conducted within a 11.5 m by 9 m room located in the Box museum, Plymouth. A 6.5 m by 3.5 m search space will be constructed in the center (Figure 1), surrounded by a 1.5 m‐high black curtain that either has a pattern resembling the night sky or is monochromatic black, depending on the condition. Two cameras accompanied by room microphones will be positioned at opposite corners to capture children's behavior. Entry into the search space will be through a centrally‐positioned opening in the curtain, revealing the array at the start of each trial. A green circular mat (approximately 0.5 m in diameter) will mark the starting point, positioned directly in front of the entrance at a distance of 0.5 m. Search locations consist of 10 small wooden boxes (16.5 cm long, 11 cm wide, 8.5 cm high) positioned on the ground within the search space and affixed using Velcro pads. These will be colored blue in one hemispace, and yellow in the other. Boxes will be 1.5 m apart, forming two symmetrical pentagons parallel to the mid‐sagittal axis. The array will remain consistent across participants. Boxes will have hinged lids with an elastic attached to the inside so that they close after being opened for inspection. A separate 2 m × 3 m “familiarity” area marked by a rectangular blanket will provide a designated space for the experimenter to interact with the participant. Target objects will be circular stickers (5 cm diameter) depicting novel objects, paired with a novel word introduced using a physical storybook (Figure 2). Participants will complete the word learning task on a handheld tablet.

### 2.3 Design and Materials

Parents will complete the Early Child Curiosity Questionnaire (ECCQ; Altmann et al. 2024), a 25‐item parental report measure designed to assess individual differences in children's trait curiosity. Responses are provided on a 7‐point likert scale (i.e., 1 = Strongly disagree; 7 = Strongly agree), and differentiate between three factors: Novelty (e.g., “My child regularly seeks out new experiences”), Investigative (e.g., “My child usually inspects objects from all angles and sides”), and Interactive (e.g., “My child typically seeks clarification for things they do not understand,” e.g., how something works) curiosity. Despite a structure supported by multiple factors, items will be averaged into a mean score to produce an overall trait curiosity score. Children will be randomly assigned to one of two conditions (i.e., HC or LC) and presented with novel word‐object pairings via a custom‐made storybook. A female experimenter will read the story in a child‐friendly manner.

The narrative, inspired by Piazza et al. (2021), follows Susie, an astronaut who lives in space and is trying to help her alien friend. This space‐themed narrative (including concepts such as planets and a rocket) was previously shared with children aged 3.5–4‐years‐old (Piazza et al. 2021) and is also common within UK book series aimed at children as young as 1 year old, including *Peppa Pig: Peppa in Space* (Peppa Pig 2019), *That's Not My Rocket…* (Watt 2019), *Where's Mr Astronaut?* (Arrhenius 2019), and *Zoom to the moon*! (Pat‐a‐Cake 2019). The story presents the reader with a problem: Susie's friend needs certain pieces (novel objects with novel names) to build something that will only be revealed at the end of the storybook. However, the pieces are scattered in space. Susie explains she can fly in her spaceship to find these pieces; she flies off to find each object, hinting that she needs our participants’ help. Participants will be asked to find a sticker of the novel object, and within the storybook, there will be a white circle where they can place it. When all the pieces (i.e., stickers) are found, it is revealed that they build a rocket, and Susie and her friend are then able to fly away in their spaceships together. To promote curiosity, the narrative introduces a knowledge gap—that is, the alien is building something, but the participant does not know what. This uncertainty is further emphasized through embedded prompts (e.g., “I wonder what we are going to build”), while the novelty of the objects is also brought to the participant's attention in the same manner. Additionally, an unexpected narrative event violates participants’ expectations between the presentation of the second and third object—that is, Susie suggests they start assembling the parts, but a new alien appears to warn her that they need to leave because of an approaching storm. In addition to the story, the environment will also be decorated with a moderate amount of visual complexity. The LC condition will present the same novel words and objects in a storybook format, but without any accompanying narrative. Instead, participants will see the novel objects against a black background in identical positions to the HC condition, and the same sentence frames will be used to introduce the words (Figure 2).

**FIGURE 3:**
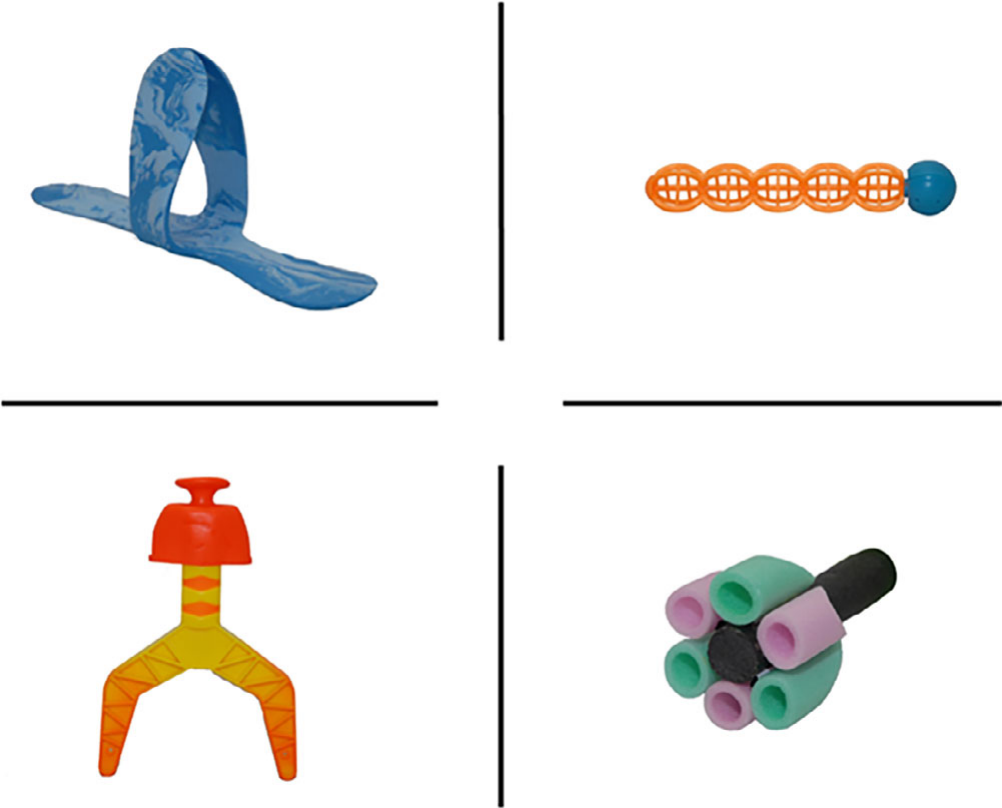
Novel objects.

Participants in both conditions will be exposed to the same four novel words (wug, lif, neem, and pok; Kalashnikova et al. 2018). Each word is randomly paired with a novel object (Figure 3) from the NOUN database (Horst and Hout 2016), which were selected based on their high novelty scores (91%–94%). Participants will hear each of the novel words four times in each of the following sentence frames: “The alien said it is called a pok” (HC), “The first piece we need is a pok. What does it look like?” (LC), “This is the pok. Can you find the pok?” (both conditions) and “Yay we found the pok” (both conditions), and two extra times produced naturally by the experimenter during the search task (e.g., “Where could the pok be?”). Thus, children will be exposed to each novel word a total of six times in both conditions. The introduction of a new novel object within the story will provide a cue for the child to enter the search space and search for the novel object in sticker format. Participants will be told to “search the planet for the part” and will walk or crawl to each box, physically inspecting them by opening the lid. During the experimental trials, targets will always be present but only within the cued hemispace, counterbalanced between participants (i.e., targets will only be found within either the left or right hemispace and target locations will be either blue or yellow boxes). To account for word order effects, children in each condition will be assigned one version of the storybook out of the possible four that differ by the object presentation order. Some images in the book (i.e., the alien characters and the rocket combining all novel parts) were generated using ChatGPT (OpenAI 2025).

### 2.4 Procedure

Before the experiment, participants will take part in a 5‐min familiarization period with the experimenter to explore the environment and build rapport. The experimenter will also demonstrate how to open two sample boxes (one containing an example sticker) and encourage the child to do the same. During this time the caregiver(s) will be given consent forms and the ECCQ questionnaire to complete (Altmann et al. 2024). Caregivers will remain in close proximity but will be asked to refrain from intervening unless the child shows any signs of distress and discomfort.

The experimenter will begin the task by introducing the storybook and will prompt the participant to search for the target stickers. After the presentation of each target object, participants will be directed to the entrance of the search space, the curtain will be divided, and they will be told they can begin searching for the target. The experimenter will repeat the novel word twice to prompt the child to search for the sticker, for example, “Where could the pok be?” “Can you find the pok?” Only a single target (i.e., sticker) will be present in the array at any one time. Participants will be given 5 min to locate it (i.e., 30 s per location), during which only the child will be permitted to enter the search space. The experimenter will offer verbal encouragement (i.e., “You can't find it?” “Where do you think it is?”). Once the participant has located the hidden sticker, they will be instructed to return to the experimenter and add the sticker to the storybook in a predefined location (Figure 2). This process will be repeated for a total of four search trials, one for each novel object. If a participant fails to locate the sticker within the allocated time frame, the experimenter will show them the location of the target and allow them to place it inside the storybook. They will then begin the next phase of the storybook. Participants who fail to locate a minimum of three stickers will be excluded from the analysis (See Table 1); these children will be able to finish the experiment with the help of the researchers and will receive a gift.

**TABLE 1:** Exclusion criteria.

Participant‐level exclusions (behavioral)	Trial‐level exclusions	Participant‐level exclusions (task‐specific)
Incidents occurring during testing will be systematically recorded. Participants will be excluded if they: ● Terminate the task prematurely (i.e., refuse to complete ≥50% of trials). ● Showed sustained disengagement, defined as repeated refusal to respond across consecutive trials (≥3 in a row). ● External influence, defined as a caregiver or experimenter's interference in the tasks affecting ≥25% of trials (e.g., prompting, pointing, or guiding responses). Technical issues, defined as technical failures specific to the tablet or incorrect trial presentation.	For the word learning tablet task, accuracy will be coded on a trial‐by‐trial basis (1 = correct target selection; 0 = distractor selection). Trials with no response will be coded as NA and excluded from analyses. For reaction time (RT) analyses, only correct trials will be included. Trials in which response times exceed 2 *SD* above the participant's mean will be excluded (Ackermann et al. 2020).	Participants will be excluded from the word learning tablet task if they demonstrate: ● Consistent inattentiveness, operationalized as leaving the testing area or being off‐task for more than 50% of trials. ● A side bias, defined as selecting the same response location on ≥7 out of 8 trials (Ackermann et al. 2020). For the search task, participants who fail to locate at least 3 stickers will be excluded from analyses.

*Note*: A comprehensive table documenting all excluded participants, and the reasons for their exclusion, will be included on the OSF page.

After the final trial, but before the object being built is revealed to participants (HC), the story will indicate to the participants that they either need another piece to help the alien build (HC), or that there is still another item that needs to be found (LC). Participants in both the HC and LC conditions will be instructed to search the array for a final target but, on this occasion, no target will be present. No encouragement will be given during this portion of the task. The trial will end once the child leaves the designated search space and they will then be presented with a small alien toy, representing the alien builder in the HC storybook. Children will then finish the storybook, and those in the HC will discover they were building a rocket ship for Suzie's alien friend. At the end of the experiment, participants in both conditions will also be asked to rate how much they wanted to find the stickers by selecting one of five faces ranging from very happy (i.e., 5) to very sad (i.e., 1). Prior to asking the question, children will complete a brief training phase with the experimenter to familiarize them with the picture rating scale. Following procedures used in prior work (Ruzek et al. 2020), the experimenter will point to each face and explain its meaning. Children will then complete two practice items using familiar everyday examples (“How much do you like ice cream?” “How much do you like playing games?”), responding by pointing to the scale. If children do not respond or appear confused, the two practice trials will be repeated. This approach is consistent with evidence that when assessing children's motivational constructs, they can provide reliable self‐reports when supported by simplified response formats and visual scales (Hutchins and Jirout 2025; Ruzek et al. 2020).

#### 2.4.1 Word Learning Task

Following the story reading and search task, children will participate in a word learning task inspired by Ackermann et al. (2020). This will be administered on a touchscreen tablet held by the experimenter, an appropriate method for the sample age group (Aziz et al. 2013; Frank et al. 2016). The test will begin with a familiarization phase followed by a Four Alternative Forced‐choice test (4AFC).

**FIGURE 4:**
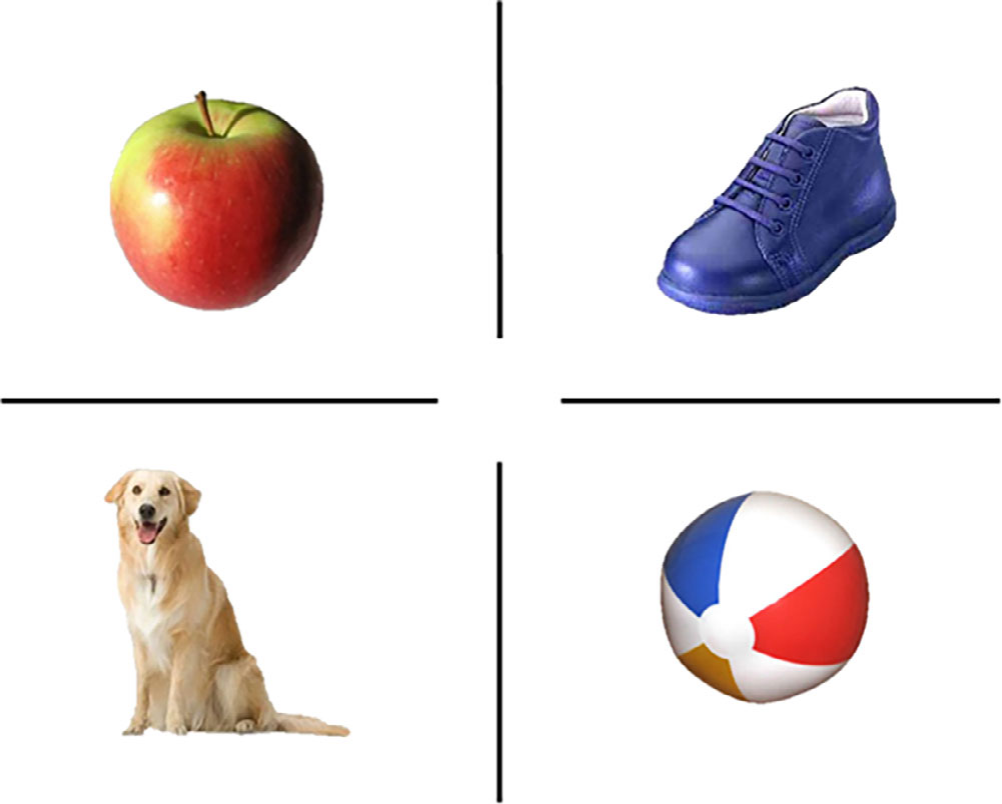
Images to be used in the familiarization trials.

The familiarization trials (*n* = 2) will present participants with images of four objects (Figure 4) that should be known to at least 82% of children learning British English by 25 months (Wordbank data; Frank et al. 2017). Images will be presented all together (e.g., Figure 4) in a 2 × 2 grid and participants will be prompted to find the object with two pre‐recorded questions: “Can you tap the dog?” and “Where is the dog?” If after two familiarization trials, the child still does not follow the tapping procedure, we will rerun both trials until they show an understanding of the procedure exemplified by correctly tapping the target object. However, we do not predict specific issues here given previous findings showing children's competence at completing such tasks (Ackermann et al. 2020). Once we are confident the child understands the task, the test phase will begin.

The test phase will include eight 4AFC trials, during which participants will be asked to tap the target novel object from a 2 × 2 grid; targets are novel objects that were paired with novel labels in the book. Each target will be presented alongside all three other novel objects from the story book, which act as foils. Pre‐recorded prompts (i.e., “Can you tap the Wug?” and “Where is the Wug?”) will be played at the start of each trial. All audio recordings were made by a female native speaker of British English (dialect details blinded for review) who spoke in a child‐friendly manner.

Image presentation will be counterbalanced so that all novel object pairings and positions occur equally. No more than two consecutive targets will appear in the same location; each target will appear twice per quadrant (See example list on the OSF). Each image will appear in a square with black borders to indicate distinct tapping areas.

Participants will have unlimited time to respond (Ackermann et al. 2020); however, tapping will be disabled for the first 3000 ms until both recorded questions are finished playing. After the response, an attention‐getter will be played for 1500 ms (a multicolored moving circle with a chiming sound; Frank et al. 2020). Participant accuracy (i.e., tapping the target or a distractor) and response time (i.e., the latency between presentation of the target word and a tap on one of the stimulus images) will be recorded.

### 2.5 Analysis

All pre‐registered linear mixed models are the maximal models that are justified based on our design and our research questions; if these models do not converge then we will minimize them according to the guidelines set forth in the literature (Barr et al. 2013). All models will be fitted using the lme4 package in R (Bates et al. 2015). To test the effectiveness of our curiosity manipulation and prior to testing our hypotheses, we will conduct independent samples *t*‐tests to examine whether there is a difference between conditions in children's interest in the task (i.e., via their own report: a score from 1 to 5) and their task engagement (i.e., the average number of inspections per minute). If our curiosity manipulation is effective, children in the HC condition should report higher interest scores and conduct more inspections per minute.

#### 2.5.1 Word Learning Task

We will first assess participants’ engagement by counting their attempts to make a response by either tapping the target or distractor image (i.e., total number of tapping responses). Previous work using touch‐screen devices shows that even 18‐ to 20‐month‐olds attempt to respond to ∼80% of the trials they are presented with (Lo et al. 2021). Consequently, we anticipate our participants will attempt all eight trials. Exclusion criteria are summarized in Table 1. Response time analyses will be conducted to index processing efficiency and engagement, with faster responses reflecting more efficient retrieval of word‐object mappings.

### 2.6 Proposed Statistical Analysis

First, to measure whether children successfully learn the novel words, we will conduct a one‐sample *t*‐test of the accuracy scores against chance (0.25). If children succeed in learning the novel word‐object associations, this should be reflected in higher than chance accuracy scores. If such a difference is not found, then we will conclude that our experimental paradigm did not provide evidence for word learning.

Analyses are based on previous experiments that used touchscreen word‐learning tasks (Ackermann et al. 2020; Lo et al. 2021). Two linear mixed effect models will be fitted to examine the effects of Age (continuous variable), Condition (HC or LC), trait curiosity (as measured by the ECCQ), and their interactions on task performance. The first model will use Accuracy as the outcome variable and will be fitted as a binomial Generalized linear mixed model. Accuracy will be coded on a trial‐by‐trial basis: with a score of 1 for correct target taps and 0 for distractor taps, while nonresponses will be coded as NA and excluded from the analyses (Table 2). A second model will include Response Time as the outcome variable and will be fitted as a Linear Mixed Model. Incorrect trials will be excluded (Table 2). Models will include random intercepts for subject and item (model syntax: Outcome Variable ∼ Condition * Trait Curiosity + Condition * Age + (1 | Subject) + (1 | Trial)).

**TABLE 2:** Summary of the design.

Question	Hypothesis	Analysis plan	Interpretations
Do higher levels of state‐induced curiosity improve word learning outcomes, and is this moderated by age and/or trait curiosity?	Compared with the LC condition, children in the HC condition will show higher accuracy scores and reduced response times in the word learning task. This effect will be moderated by age (older children show stronger learning effects) and trait curiosity (higher trait curiosity amplifies the HC benefit).	**Primary model (Accuracy)**: Binomial GLMM Accuracy ∼ Condition * Age + Condition * Trait Curiosity + (1| Subject) + (1 | Trial) **Secondary model (Response Time)**: LMM (correct trials only) RT ∼ Condition * Age + Condition * Trait Curiosity + (1| Subject) + (1 | Item) **Preliminary check**: One‐sample *t*‐test against chance (0.25) to confirm learning.	**Task validity** Accuracy > chance → word learning established Accuracy = chance → hypotheses not testable **Effect of state curiosity** HC > LC (accuracy) → curiosity enhances word learning HC > LC (SOM/path efficiency) → curiosity enhances adaptive search No significant condition effect → learning/search independent of state curiosity **Mechanistic link** SOM ↑ → accuracy ↑ → adaptive search supports word learning SOM × condition → search matters more under high curiosity No SOM–accuracy link → partially independent processes **Moderation** Condition × age → developmental variation Condition × trait curiosity → dispositional modulation Age × SOM (or 3‐way) → developmental differences in mechanism **Non‐convergent outcomes for Accuracy vs. Response Time in the word learning task** Accuracy ↑ + RT ↓ → strongest evidence of improved learning and processing efficiency Accuracy ↑ + RT ↔/↑ → improved learning with increased processing demands Accuracy ↔ + RT ↓ → faster responding without learning gains; may reflect task familiarity or response strategies Accuracy ↓ + RT ↓ → speed–accuracy trade‐off; reduced processing depth
Do higher levels of state‐induced curiosity lead to more efficient search behavior, and is this moderated by age and/or trait curiosity?	Compared with the LC condition, children in the HC condition will demonstrate shorter path lengths, higher SOM scores, and greater sensitivity to the statistical contingency across trials. This may vary by age and trait curiosity (higher trait curiosity amplifies the HC benefit).	**Primary model (Path Length/SOM across trials)**: LMM Outcome ∼ Trial Number * Condition + Condition * Age + Condition * Trait Curiosity + (1 + Trial Number | Subject) **Final target‐absent trial model**: SOM ∼ Condition * Age + Condition * Trait Curiosity + (1 | Subject) **Supplementary analyses**: One‐sample *t*‐test (initial hemispace inspection vs. 0.5) Within‐subject *t*‐test (time spent in the cued vs. uncued hemispace) Within‐subject *t*‐test (the number of inspections made in the cued vs. uncued hemispace)	
To what extent is search efficiency associated with word‐learning outcomes, and is this association moderated by higher levels of state‐induced curiosity and age?	More optimal search behavior (higher SOM) will predict greater word‐learning accuracy. This association will be stronger in the HC condition as opposed to the LC condition, and may vary with age.	**Model across learning trials**: Binomial GLMM 4AFC Item Accuracy ∼ SOM * Trial Number * Condition + (1 + Trial Number | Subject) + (1 | Item). **Final trial model**: Accuracy ∼ SOM * Condition * Age + (1 | Subject)	

*Note*: **↑** means “higher,” **→** means “leads to,” ↔ means not significant.

Successful manipulation of state curiosity will be reflected in higher accuracy scores in the HC condition compared to the LC condition. If trait curiosity and age moderate the impact state curiosity has on word learning, a significant interaction would be expected between these variables and condition (HC, LC).

#### 2.6.1 Search Task

**FIGURE 5:**
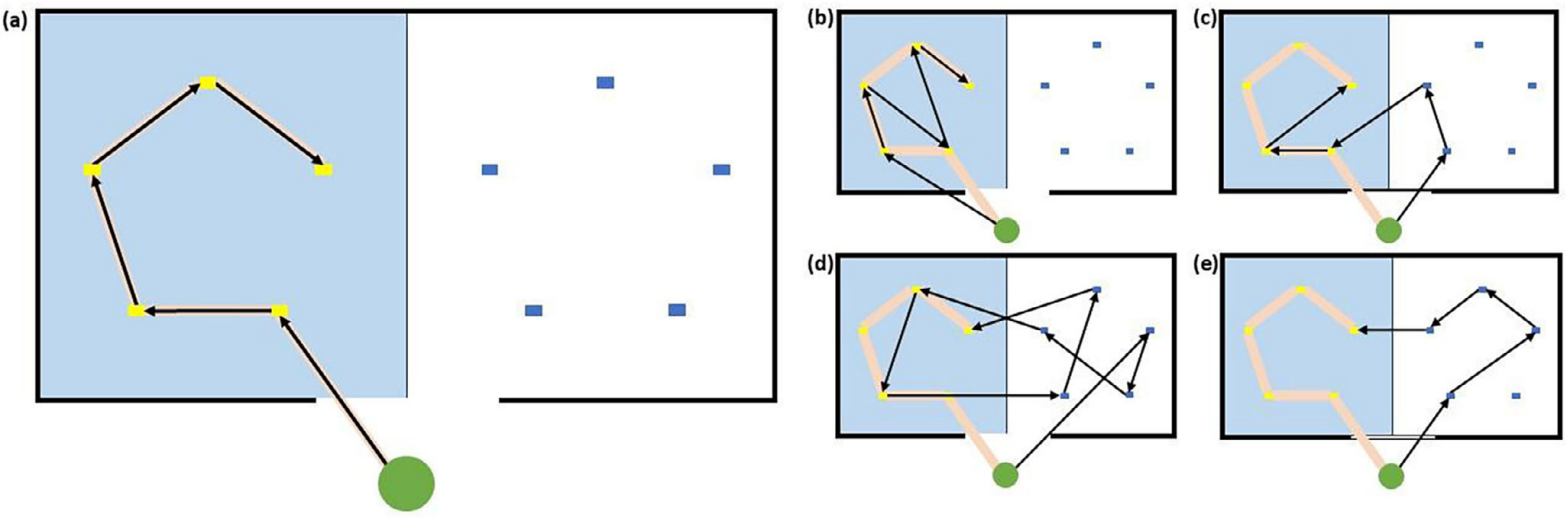
An optimal path through the array. The light orange line represents the optimal path through the experimental array when the cued hemispace is on the left side of the mid‐sagittal axis, as defined by the traveling salesman package. The blue half represents the side containing the target on each trial (i.e., the cued hemispace). For half of the participants, the figure will be inverted, with the “cued” region on the other half of the array. The optimality metric for each example above are as follows: (a) 1, (b) 0.83, (c) 0.67, (d) 0.33, and (e) 0.33.

Adapting approaches from previous studies (Baxter and Smith 2020; Dordoy et al. 2026; Pellicano et al. 2011; Smith et al. 2010), Path Length (i.e., the total Euclidean distance across inspected locations) will be used to assess whether participants respond to the probability cue. Search paths will be compared to an optimal path generated by the Traveling Salesman Problem (TSP) R‐package (Hahsler and Hornik 2007), using the MATLAB ScanMatch package (Cristino et al. 2010). An optimal path is defined as the shortest possible route through the array to the target, prioritizing the “cued” hemispace (See Figure 5). A Search Optimality Metric (SOM) will be produced, varying from 0 to 1, where higher values indicate more efficient search paths. During the final target‐absent trial, the aforementioned dependent variables will be calculated, although the SOM score will only examine the initial 10 inspections and will be compared to an optimal path through the entire array. This approach captures sensitivity to the probability cue and participant search optimality, while excluding re‐inspections that would reflect attempts to verify the target's absence after all locations have already been inspected.

### 2.7 Proposed Statistical Analysis

To examine whether participants adjust their search strategy in response to the statistical contingency, a Linear Mixed Model will assess whether Path Length and SOM are predicted by Trial Number, Age, trait curiosity and Condition. Each participant will contribute four data points (i.e., one for each trial; model syntax: Outcome Variable ∼ Trial Number * Condition + Condition * Age + Condition * Trait Curiosity + (1 + Trial Number | Subject). If participants are able to adjust their search behavior in response to the statistical contingency, search paths will become more optimal as trials progress (i.e., higher SOM scores). Additionally, if state curiosity is successfully manipulated and improves search behavior in response to the statistical contingency, participants in the HC condition should have shorter path lengths and more optimal search in comparison to the LC condition. A third model will be fitted including SOM during the final target‐absent trial as the Outcome Variable (model syntax: Outcome Variable ∼ Condition * Trait Curiosity + Condition * Age + (1 | Subject)). If state curiosity has facilitated learning of the statistical contingency, participant search paths will be more optimal in the HC compared to the LC condition and if trait curiosity, state curiosity, or age moderates relationships in any of the three models, there will be significant interaction between the factors. To account for the possibility that participants will learn that targets do not appear in the same box more than once, the models including SOM as the outcome variable will be repeated, redefining the optimal path to exclude locations that previously contained a target. Model structure and interpretation of dependent variables will be the same.

In addition to the proposed models, whether participants inspected the cued hemispace first will be recorded on each trial (i.e., 1/0) and entered into a one‐sample *t*‐test against a chance level of 0.5. If search decisions are guided by the statistical contingency, participants will be expected to direct initial inspections toward the cued hemispace. Additionally, during the final target‐absent trial, the amount of time spent and the number of locations inspected in each hemispace, will be compared using a within‐subjects *t*‐test. If participants learn the statistical contingency, this should be reflected in greater time spent and more locations inspected in the cued hemispace. A Pearson's correlation is also expected to confirm a strong correlation between Path Length and SOM (e.g., Dordoy et al. 2026).

#### 2.7.1 Combined Analysis of Word Learning and Search

Two final binomial Generalized linear mixed models will explore whether participant search strategy is predictive of word learning. The first model will include item accuracy on the forced choice task as the outcome variable and Trial Number, SOM, Condition and their interactions will be entered as fixed‐effects (model syntax: 4AFC Item Accuracy ∼ SOM * Trial Number * Condition + (1 + Trial Number | Subject) + (1 | Item). A significant SOM × Trial Number × Condition interaction would indicate that the association between word learning and search optimality changes across trials differently between conditions. More specifically this association would be supported with increasingly optimal search supporting word learning in the HC condition. Trial Number refers to the order in which an item (novel word‐object association) was presented during the search task. Because each item is tested twice in the forced‐choice task, accuracy is modeled at the item‐response level, with repeated observations per item.

A second model will focus on the final, target‐absent trial. Accuracy on the word learning task will be the outcome variable alongside SOM, Age and Condition as fixed‐effects (model syntax: 4AFC Accuracy Score ∼ SOM * Condition * Age + (1 | Subject)). If adapting to environmental regularities supports word learning, higher SOM scores will predict greater word learning accuracy. Moreover, if the state curiosity manipulation facilitates this, it will be reflected in an interaction with condition. A summary of our analysis plan and design is in Table 2.

## Author Contributions

**Rana Abu‐zhaya**: supervision, writing – review and editing, conceptualization. **Shaun Dordoy**: writing –review and editing, writing – original draft, conceptualization, investigation, methodology, software, visualization. **Alastair Smith**: supervision, writing – review and editing, conceptualization. **Anna Caunt**: writing – original draft, writing – review and editing, methodology, conceptualization, investigation, software, visualization.

## Funding

The authors have nothing to report.

## Conflicts of Interest

The authors declare no conflicts of interest.

## Data Availability

The data that support the findings of this study are openly available in Navigating Knowledge: Effects of State‐Induced Curiosity on at https://osf.io/5bmhk/?view_only=dc3d7975debb4c2bbfa6060d428f373a.
